# Concerning the Present Standing of the Water Treatment of Typhoid Fever

**Published:** 1887-05

**Authors:** Ernst Brand

**Affiliations:** Stettin, Germany


CONCERNING THE PRESENT STANDING OF THE WATER TREATMENT OF TYPHOID FEVER. *By* DR. ERNST BRAND, Stettin, Germany. (*In Deutsche Medicinische Wochenschrift, Nos. 1 to 11 inclusive.*)

In the *Berliner Klinische Wochenschrift*, of November 9th, 1885, Senator of Berlin said that the so-called water treatment, or "method of Brand," of typhoid fever, was not only not in use in any Berlin hospital, but also that as a method of treatment it produces no conclusive results. It has been condemned also by Leyden of Berlin, Gläser of Hamburg, and Ebstein of Göttingen. The *Gazette Hebdomadaire*, of January 15th, says, "The method of Brand in the treatment of typhoid fever has fallen into disuse among the German physicians." The author thinks all this calls for a defense of the treatment assailed, and he proposes to "break a lance" with the gentlemen named in behalf of his method.

The articles contributed are so voluminous and the style so discursive that great condensation is necessary in this abstract, but an earnest attempt is made not to omit any matter essential to the argument of the author.

"The ground upon which the gentlemen named base their conclusion is that, according to their experience, the very troublesome treatment by baths gives no better results than the expectant treatment gives. Ebstein (*Die Behandlung des Unterleibstyphus*, Wiesbaden, 1885,) with the expectant treatment lost 13 in 225 cases, or 5.5 per cent. Gläser

(*Berliner Klinische Wochenschrift*, 1885, No. 12) reports from the Hamburg hospital a mortality of 7 per cent. with expectant, and of 8 per cent. with the water treatment, and (*Deutsche Medicinische Wochenschrift*, 1885, No. 12) 102 cases treated "without water or other antipyretics" with 3 deaths.

Senator (*Berliner Klinische Wochenschrift*, 1885, No. 45) reports the mortality in the Augusta hospital with the expectant treatment, without baths or antipyretics, as 12.3 per cent.; and in the Bethany hospital, and in the Moabite military hospital, with energetic use of water and antipyretics, as 13.5 per cent. and 15.2 per. cent. respectively.

Under the expectant method the following mortality occurred, viz., With Liebermeister, 27.3; with Griesinger, 18.9; in Kiel, 15.6; in Leipzig, 18.5; in Vienna hospitals, over 20; in Dresden, 13; in Strasburg, 23; in Paris 32; and during the siege of 1870–71, 60.8 per cent.

The author claims that the expectant method remains what it always has been, and he gives the following from Ebstein as illustrative: "Calomel as abortive treatment; against the development of typhoid, careful diet, good surroundings, cleanliness, fresh air, wine, hydrochloric acid, with and without quinia; against the temperature, accompanied by grave brain or heart symptoms, salicylic acid; against heart failure, wine, cognac, camphor; against bronchial and lung affections, 'Flores benzoës' and preparations of ammonia; against the stupor, musk, cold frictions, and an ice bag to the head." Ebstein's diet list comprises milk, meat broths, meat extracts, yolk of the egg, liqueurs, coffee and spirits.

"In Hamburg under the expectant treatment the mortality in typhoid fever was 19 per cent., and under the administration of Gläser it has recently fallen, under both expectant and so-called water treatment—both with and without medicine, to 3.2 to 20 per cent. In Göttingen, prior to 1861, under expectant treatment it was 25 to 33.3 per cent. Under timid water treatment—one bath daily—it ranged from 3.2 to 20.2 per cent.; and now under Ebstein's expectant treatment it stands at 5.5 per cent."

The author claims that after a careful examination of the communications of Gläser and Ebstein, since the surroundings and service are the same as formerly, one is forced to the conclusion that the lessened mortality reported by these gentlemen is due simply to their increased skill in the management of the expectant method. Ebstein reports 222 cases of recovery as of great or moderate severity, and of these, intestinal hemorrhage in 4.9 per cent. against 5.6 per cent. formerly; pneumonia in 2.7 per cent. against 20 per cent. formerly; ulceration from pressure of decubitus, 1.8 per cent. against 10 per cent. formerly. The average duration of the stay in the hospital was twenty-nine days, and during the seven years from 1877 to 1884 there was no death from typhoid fever in the Policlinic at Göttingen.

Gläser replies in a somewhat irritated tone to the remark, by Mayer in Aachen, that typhoid fever in Hamburg is of mild variety, and reports a series of 102 cases with their complications, treated "without water or other antipyretic measures."

"In the following table I arrange these cases and remarks upon the complications in parallel columns:

| *Gläser's 102 cases.* | *Comments.* |
| --- | --- |
| 1. Average stay in the hospital, 49 days. Average duration of the fever, 18 days. | 1. Both stand in marked contradiction. The duration of the fever is that of light cases (sixteen days according to Jürgensen); the stay in the hospital is that of severe cases (forty-four days is the average in military hospitals; thirty-seven that of clinical institutions). |
| 2. A temperature above 41° C. (105.8° F.) twice, or 2 per cent. | 2. In 100 of my non-selected case-histories I find 22 with a temperature over 41° C. (105.8° F.), 3 over 42° C. (107.6° F.), without death or other significant result. |
| 3. A pulse of 144, 5 times. | 3. A common occurrence in nervous anaemic and protracted cases of typhoid fever. |
| 4. Ulceration from pressure of decubitus, 1 per cent. | 4. Formerly 9 per cent. |
| 5. Recurrence, 1 per cent. | 5. Formerly 8.6 per cent. |
| 6. Pneumonia, 11 per cent. | 6. Formerly 20 per cent. |
| 7. Pleuritis, 3 per cent. | 7. Formerly 3.6 per cent. |
| 8. Intestinal hemorrhage, 6 per cent. | 8. Formerly 7.3 per cent. |
| 9. Profuse diarrhœa 3 times, and tympanites 2 times. | 9. In every case of developed typhoid tympanites is never absent, and profuse diarrhœa is a common complication. |

In the light of such statements it must be admitted that the Hamburg cases are light ones. This conclusion gains additional confirmation from Gläser's statement that his typhoid cases have "'quiet-blessed' sleep," for it is true the world over that under expectant treatment, stupor, somnolence, coma or delirium or phantasy are common.

What Gläser and Ebstein call energetic hydrotherapy is by no means such. According to Gläser it consists in the administration of baths at 16° R.; (60.8° F.) during the time from 7 A. M. to 9 A. M., whenever the temperature of the body reaches 39° cr 39.5° C. (102° to 103°), no baths at night. At Göttingen baths of ten minutes' duration from 26° to 28° R. The number of the baths during the entire course of the fever was, 1860–1863, to each patient, 8 to 10; 1863–1868, 15; 1868–1872, 30 to 35; that is, not more than one bath daily at any time. In the Moabite (Berlin) military hospital, together with quinine and other antipyretics, baths at 22° R., cooled to 18° R., continued 10 to 15 minutes. In cases of children and those of feeble constitutions, 27° R. cooled to 20° R. Never more than 4 baths in the 24 hours, and this only in cases of specially high temperatures, otherwise 1 to 3 daily. In the Bethany hospital (Berlin), in cases of temperatures of 39.5° C. (103° F.) ice bags are applied to the head, and ten-minute baths at 24 to 20° R. are given 3 or 4 times daily, none at night.

For the expectant treatment Murchison gives a long list of indications; for the "water treatment" there is only one, viz., to prevent and reduce every exacerbation of the fever from the beginning to the end of the fever, day and night. This indication announced by me years ago obtains to-day.

The following arrangement in parallel columns will show the difference between the so-called antipyretic and hydrotherapeutic methods of treatment:

| *Hydrotherapeutic.* | *Antipyretic.* |
|---|---|
| 1. The severe cases of typhoid will be changed in all respects to lighter ones, and those of mild grade become less severe. | 1. A change into a typhoid case free from fever, i. e. without temperature increase, without regard to functional disturbance. |
| 2. *This happens*— | 2. *This happens*— |
| *a.* Through prevention of every increase of temperature, and the gradual establishment of a temperature near the normal. | *a.* Through diminution of excessively high temperatures. |
| *b.* Through prevention of functional disturbances of the organs. | *b.* Through a change from continued and subcontinued fever to a remittent type. |
| *c.* Through a diminution and suppression of the process. | |
| *d.* Through a preservation from complications. | |
| 3. Every 3 hours a bath at 15° R. for 15 minutes, and whenever the temerature is above 39° C. (102°.2 F.). | 3. *Formerly*, baths whenever body temperature was 40° C. (104° F.), and every other evening an antipyretic. *Now* an antipyretic in the evening, and during the night baths frequently enough to keep the temperature nearly normal; during the day nothing, or at lease a bath only in cases of unusually high temperature. |
| 4. The treatment is directed against the process as a whole. | 4. The treatment is directed against one symptom, the fever. |

| | |
|---|---|
| 5. Baths are used exclusively. | 5. Baths and antipyretics are used. |
| 6. Mortality.<br>When the treatment is begun early and regularly carried out the mortality will be:<br>In family practice—1 per cent.<br>In general practice—3 to 4 per cent.<br>In military hospitals—4 per cent.<br>In general hospitals—5 per cent.<br>without special change. | 6. Formerly 0 to 23 per cent. with an average of 7 to 8 per cent.<br>Now, 10 to 18 per cent., with marked diminutions. |

One sees at a glance that the two methods have only the baths as a common feature.

The author calls attention to the fact that fifteen years ago he denied that the treatment by baths in vogue at Berlin and Hamburg had any influence upon the course of the disease or upon its mortality. He says that Gläser is correct in claiming that his (Brand's) method of hydrotherapeutic treatment gives uncertain results in mortality, but asserts that this uncertainty is inherent more in the nature of the case under treatment than upon the method employed. A list of fifty-eight communications concerning typhoid fever is given which appeared between 1878 and 1886.

Those who favor the antipyretic method claimed that the antipyretic effect is the only good thing accomplished, and the author thinks that the water treatment has consequently, because it requires so much work, fallen into disuse. He thinks this claim unsupported by facts, and proposes to answer the question, "What is the systematic hydrotherapeutic treatment of typhoid fever worth, and has it achieved what was claimed for it?"

In answer to this question the following facts are given: The cold-water treatment of Brand is in use throughout the Prussian army and the 13th (Würtemburg) army corps. The mortality in typhoid cases was: 1874, 12 per cent.; 1875, 10.9 per cent.; 1876, 10.8 per cent.; 1877, 9.8 per cent.; 1878, 8.9 per cent. The Sanitary Department reports that from 1887 to April 1, 1882, at Stralsund, under Brand's method of cold baths, only one death occurred.

The mortality in the entire German army from typhoid fever was in 1881, 8.3 per cent.; in the Italian (1874–78), 28–36.8 per cent.; in the Austrian (1873–78), 26.8 per cent.; in the French (1875–80), 36.5 per cent. Dr. Paul Helm reports concerning the treatment of typhoid fever in the military hospital at Stralsund, under Ziemsen's method (1873–77), a mortality of 10 per cent.; under Brand's method in 247 cases, only one death, and that case came under treatment on the fourteenth day of the illness.

Vogl, in Munich, says (*Deutsches Archiv für Klinsche Medicin Bd. XXXVI. Heft 5u. 6.*): "The average mortality under the Brand method of treatment is 2.7 per cent. The greatest mortality in any one year is 4.7 per cent." The following table is from Munich, where typhoid cases have been treated at different stations, according to the methods of Liebermeister and Brand, but with other conditions as nearly the same as possible:

| PERIOD. | NO. OF CASES. | PERCENTAGE MORTALITY. | TREATMENT. |
|---|---|---|---|
| 1875- 6 | 76 | 15.8 | Liebermeister. |
| | 66 | 4.5 | Brand. |
| 1876- 7 | 194 | 6.7 | Liebermeister. |
| | 141 | 3.5 | Brand. |
| 1877- 8 | 77 | 3.8 | Liebermeister. |
| | 56 | 0.0 | Brand. |
| 1878- 9 | 115 | 6.1 | Liebermeister. |
| | 92 | 15.2 | |
| 1879-80 | 110 | 10.8 | Liebermeister. |
| | 98 | 3.9 | Brand. |
| 1880- 1 | 16 | 18.8 | Liebermeister. |
| | 25 | 4.0 | Brand. |
| 1881- 2 | 22 | 9.1 | Liebermeister. |
| | 42 | 4.7 | Brand. |

In the total number of cases the percentage of complications under the Liebermeister treatment was 102 per cent.; under that of Brand, 65.2 per cent., as follows:

| | Brand. | Liebermeister. |
|---|---|---|
| Pleuritis | 1 | 16 |
| Delirium | 6 | 93 |
| Involuntary Evacuations | 1 | 18 |
| Bloody Stools | 2 | 21 |

Under the Brand treatment there was absolutely no pneumonia, disturbance of hearing, vomiting, stupor, muscular tremors, convulsions, suppression of urine, catarrh of the bladder, peritonitis, or perforation.

Vogl further says: "Among the thousands of baths I have never, either before, during, or after the administration of the bath, seen a single case of collapse." He says, also, that whereas formerly there were many cases of general disability following typhoid fever, since the adoption of Brand's method (1876), he can remember only three such cases. He thinks that pulmonary complications, when present, do not contra-indicate the baths. The author claims that Vogl supports him in every point which he claims for the exclusive hydro-therapeutic method.

Jürgensen is the director of the Policlinic at Tübingen. From this clinic Betz (*Ueber Typhus Abdominatis, Dissertationsschrift, Stuttgart, 1885*), reports 220 cases of typhoid fever, with 4 deaths, or 1.8 per cent. Of these fatal cases 2 had no baths and 1 was treated principally by antipyretics. Jürgensen is of the opinion that the treatment by baths retards the development of the bacillus of typhoid fever. At this clinic diarrhœa and intestinal hemorrhages are not considered contra-indications for the continuance of the cold baths. Jürgensen said at the Congress in Wiesbaden, 1882, "I think that the water treatment must be considered the best, and that no other antipyretic method can at all compare with it."

Liebermeister said (*Congress für Internal Medicin, 1885*): "Antiseptics have made an epoch in surgery and saved countless lives. I think that when the antipyretic treatment is once completely established, it will save still more lives."

Among French authors Glénard, Tripier and Bouveret are mentioned as champions of the Brand method. Tripier and Bouveret have published a book of 635 pages, entitled, "La fièvre typhoïde, traitée par les bains froids" (Paris: Bailière et fils. 1886). The observations are based upon 233 cases treated from 1874 to 1885, partly in Hôtel-Dieu, and partly in Hôpital de la Croix Rousse, in Lyons. These cases were selected from among those in the hospital as the most severe, and were treated without any medicine, and the method was at first imperfectly carried out. Under these unfavorable circumstances the mortality was 8.5 per cent. The mortality in 1882–3 was 5 per cent. The authors agree with one another on the following points: In order to get the best results the baths must be commenced upon the first day of the fever. There is no danger at all of the patients taking cold. In order to get the best results the method must be carried out in its minutest details. The author remarks that this last observation is prompted by his own experience. While it will be found that the temperature tends to rise after the bath to the former temperature, it will also be found that, after a few baths, there begins a gradual decline in the average temperature for the day.

Tripier and Bouveret found that delirious headache and mental irritability rapidly disappear. They consider the cold bath a heart tonic. They say: "The method of Brand increases the energy of the heart and the tonus of the vessels, and prevents the occurrence of paralysis. In severe cases the pulse was not above 120. Dicrotism did not appear in cases which came under treatment early in the attack, and in others it disappeared."

Tripier and Bouveret think that the influence of the cold bath is favorable in preventing and arresting bronchial catarrh and pulmonary hypostasis. In Germany pneumonia is only occasionally a complication, but in France it is the most frequent cause of death. Tripier and Bouveret consider severe pulmonary complications contra-indications for the cold baths, but stop them only when pneumonia occurs very late in the illness, and is accompanied by extreme adynamia and cardiac weakness. In this connection Bouveret refers to and confirms Jürgensen's remark, "He who has fever does not take cold." In the experience of Tripier and Bouveret diarrhœa ceased on the seventh or eighth day, and in cases treated from the beginning it did not occur; tympanites was not present, and the amount of urine was 2 to 4 times as much as it was in cases treated with medicines. Bouveret thinks that in cases in which the Brand treatment is begun before the fourth day, there will be no intestinal ulcers, and bases this opinion upon the following observations:

1. Cases treated from the beginning show absolutely no signs of ulceration.

2. The results of intestinal ulceration, hemorrhage and perforation, do not occur.

3. The statistics quoted show that intestinal hemorrhage occurs only in those cases which come under treatment late in the illness.

4. Pathological anatomy shows that the intestinal lesion by no means always goes on to ulceration, but in cases of middle grade of severity only to infiltration.

Vogl gives the following table:

| COMPLICATIONS. | METHOD OF TREATMENT. EXPECTANT. | LIEBERMEISTER. | BRAND. |
|---|---|---|---|
| Severe bronchial affections | 25.0 per cent. | 16.6 per cent. | 9.5 per cent. |
| Ulceration from decubitus | 10.0 " | 2.6 " | 2.7 " |
| Severe brain symptoms | 25.0 " | 5.2 " | 0.9 " |
| Intestinal hemorrhage | 5.6 " | 0.3 " | 0.0 " |
| Peritonitis and perforation | 9.0 " | 0.5 " | 0.0 " |

Vogl is the only one who has reported ulceration from decubitus in cases treated by the method of Brand.

He considers the cold baths contra-indicated by the following conditions:

1. Cases requiring absolute rest from peritonitis, with or without intestinal perforation, late intestinal hemorrhage, venous thrombosis—conditions, however, which he has not seen in cases treated by Brand's method.

2. Affections of the larynx and pleura Tripier and Bouveret class as primary contra-indications for the use of the cold baths:

1. Peritonitis and perforation.

2. Severe intestinal hemorrhage occurring late.

3. Pericarditis and pleuritis occurring toward the close of the illness.

They think, however, that we should endeavor to decrease, rather than increase, the number of contra-indications. The author agrees with Glénard in thinking that the baths should be continued in cases of intestinal hemorrhage in which the temperature is over 39° C. (102.° F.). "In my opinion the contra-indications are, upon the whole, simple. So long as one is not in possession of a medical procedure that gives safer results than does the water treatment, this should always be used. No obstacles should be allowed to prevent it, particularly since they are usually imaginary and their removal presents no difficulties." By way of explaining what are usually considered indications, contra-indications and conditions which are usually considered objections to the employment of cold baths, the author at this place in the discussion gives brief statements of a few cases recently encountered by him. These cases came from all classes of patients, and include intestinal hemorrhage, great cardiac weakness and pulse frequency, alcoholic excesses, poverty, and one case complicated with pregnancy, pertussis and vomiting, all making good recoveries, sound living children being born in the pregnant cases. In recapitulation, the water treatment, administered according to my directions, gives the following results:

"1. Not only the possibility of preventing each exacerbation, but also the conquering of the fever *in toto* and its causes.

"2. The prevention of functional disturbances of the brain, heart, lungs, kidneys and skin.

"3. Prevention of intestinal catarrh and the giving of complete nourishment.

"4. Prevention of intestinal ulceration in those cases treated from the inception of the attack.

"5. A prevention of complications in cases treated from the beginning, and a diminution in those coming under treatment late in the attack."

Upon the whole, under the systematic water treatment, there remains of the usual typhoid fever picture only: *a.*—A light fever; *b.*—an unimportant bronchial catarrh; *c.*—a swelling of the spleen; *d.*—the roseola; *e.*—the infiltration of the intestinal glands. All else will be controlled, prevented, and the severest case will run the course of the lighter ones when the treatment is given from the beginning.

One may compare this powerful effect of the systematic treatment of typhoid fever with cold baths with that of the Lister treatment of wounds, in securing primary union. A few years ago both were considered impossible and therefore not believed. With regard to antiseptics this is changed, but with the water treatment not completely.

The consideration of the effect upon the rate of mortality is not so pleasant, because statistics are misused and much is thus brought into contempt.

In the year 1877 I reported in the second edition of my monograph 8,141 cases treated with the hydrotherapeutic method, with a mortality of 7.4 per cent. Now the number of cases is 19,017 and the mortality 7.8 per cent.

| | CASES. | DEATHS. | PERCENTAGE. |
|---|---|---|---|
| *a.* Prior to 1877 | 8,141 | 600 | 7.4 |
| *b.* Military Hospitals— | | | |
| 1877 | 2,081 | 206 | 9.8 |
| 1878 | 2,112 | 190 | 8.9 |
| 1879 | 1,741 | 163 | 9.4 |
| 1880 | 2,534 | 226 | 8.9 |
| *c.* Military Hospitals— 2d Army Corps— | | | |
| 1882–3 | 477 | 17 | 3.5 |
| 1883–4 | 429 | 28 | 6.5 |
| 1884–5 | 392 | 14 | 3.5 |
| 1885–6 | 188 | 6 | 3.1 |
| *d.* Jürgensen (Tübingen) | 220 | 4 | 1.8 |
| *e.* Vogl (Munich) | 221 | 6 | 2.7 |
| *f.* Tripier and Bouveret | 481 | 29 | 6. |
| | 19,017 | 1,489 | 7.8 |

By the introduction of statistics from Liebermeister, Ziemsen, Hagenbach and others, this number might be considerably increased, but they all use antipyretics, and the number is already large enough for the purposes of this article. It must be remembered that these 19,017 cases include all primary and secondary complications, all ages, both sexes, epidemic and sporadic, benign and malignant cases, hospital and private practice, and that of civil and military, of war and peace, and all circumstances which modify the nature and course of typhoid fever. And the treatment, too, while in the main the employment of cold baths, is not the method of Brand, but rather such modifications in each case as suited the individual clinician reporting. The following cases, which are available for use, are partly from my monograph, and partly new:

| | CASE. | DEATHS. | MORTALITY |
|---|---|---|---|
| 1. Wiedner | 20 | 00 | 0.0 per cent. |
| 2. Winternitz | 86 | 2 | 2.3 " " |
| 3. Cayla | 63 | 4 | 6.3 " " |
| 4. Jürgensen (Kiel) | 160 | 5 | 3.1 " " |
| 5. Opitz | 66 | 0 | 0.0 " " |
| 6. Scholz | 125 | 5 | 4.0 " " |
| 7. Popper | 20 | 1 | 5.0 " " |
| 8. Riegel | 156 | 7 | 4 4 " " |
| 9. Stöhr | 120 | 8 | 6.6 " " |
| 10. Leichtenstern | 373 | 21 | 5.4 " " |
| 11. Rollet | 126 | 4 | 3.1 " " |
| 12. Hayfelder | 21 | 0 | 0.0 " " |
| 13. Pfeifer | 58 | 3 | 5.2 " " |
| 14. Jürgensen (Tübingen) | 220 | 4 | 1.8 " " |
| 15. Tripier and Bouveret | 233 | 20 | 8.5 " " |
| 16. Ballivet (Thoiry) | 73 | 3 | 4 1 " " |
| 17. Rondet and Grabinisky (Curis) | 11 | 0 | 0.0 " " |
| 18. " " " (St. Germain) | 33 | 0 | 0.0 " " |
| 19. " " " (Neuville) | 122 | 6 | 4.9 " " |
| 20. Glénard | 56 | 1 | 0 5 " " |
| 21. Vogl | 221 | 6 | 2.7 " " |
| 22. Garison Hospital II. Army Corps, 1882-5 | 2711 | 117 | 4.3 " " |
| 23. Brand (entire practice) | 479 | 17 | 3.5 " " |
| | 5573 | 234 | 3.9 per cent. |

A few of Jürgensen's cases reported above may have had occasionally other treatment in addition to the baths, but all of the others had systematic water treatment. Hence we may say that the mortality is from 4 to 8 per cent. instead of 16 to 20 per cent. under the expectant treatment. In this table the mortality above 4 per cent. is not due to the inefficiency of the hydrotherapeutic treatment, but to special circumstances. To illustrate, Tripier and Bouveret report that during the first two years they subjected only the most severe cases to the water treatment, and also did not carry it out fully. They say that they will not again have to report a mortality greater than 5 per cent., and in fact M. Tessier (*Lyons Medical*, *No. 32*) reports 139 cases with 7 deaths, or 5 per cent. In my opinion the mortality at Lyons will be still lower if they will diminish the number of contra indications, and will treat the complicated and severe cases according to my plan. There is always a risk, since there is occasionally a case which will prove fatal if the treatment is not begun at the beginning of the attack.

Popper, Stöhr, and Leichtenstern report a mortality over 4 per cent., but their statistics date from a time when the treatment and nourishment were not so precise as they have been since 1877.

The mortality of the second army corps is 4.3 per cent., but for the entire German army for the same period it is 8.3 per cent. In the second army corps the diagnosis of typhoid fever is never made, unless, in addition to the characteristic symptoms, the fever lasts sixteen days, and cases are not considered severe unless the fever lasts twenty-one days and is over 40°C. (104°F.). In this corps the mortality from 1882 to 1886 ranged as follows: 1882–3, 3.56 per cent.; 1883–4, 6.53 per cent.; 1884–5, 3.57 per cent.; 1885–6, 3.19 per cent. A basis for the statement that a morality of 4 per cent. is too high lies in the fact that statistics are incorrect, many of the cases reported either never having had a drop of water used in the treatment or having died before the use of the baths had time to produce its characteristic effect. Numbers 2 and 10 in my own death list belong in this category. No. 2 had received only one bath, and No. 10 died under the care of another physician during my absence.

Of Jürgensen's 4 fatal cases 3 were not bathed at all, and among the 6 deaths in the Pomeranian military hospital (1885–6) 2 were treated exclusively with antipyrin. One must not forget that under any method of treatment a very great number of cases of typhoid fever in succession will recover, and consequently one must avoid the *post hoc ergo propter hoc* fallacy.

In those cases in which a predisposition to complications exists, or in which indeed the complications already exist, the treatment has nothing whatever to do with the typhoid fever, but rather with the croupous, diphtheritic, pyæmic or septic processes, all of which have special indications of their own. I want to make this condition clear, since otherwise an intelligent judgment of the water treatment cannot be arrived at. The idea that complicated and degenerated (*sic*) cases are the ones with which to test the treatment is incorrect, for with such cases a special treatment for the typhoid fever has nothing to do. That in such doubtful cases the water treatment, according to other indications, may be of assistance is a topic for separate consideration, and is discussed in my monograph.

The establishment of a light course in every case of typhoid fever is the object of the systematic water treatment, and that no death occurs is the proof that the object is accomplished. In my opinion that is a demonstration so safe and radical that no one can have aught against it. It is to be expected that one may say, "You go too far," but on the contrary we can not go too far in this treatment of typhoid fever.

In the use of statistics in this connection it is necessary:

1. That the number of observed cases must be as great as possible, including many hundred.

2. That they must be included in a period of many years.

3. That both sexes, all ages, social conditions, constitutions and temperaments, must be included.

4. That so far as possible all primary complications must be present.

5. That the cases must come under treatment at the beginning of the attack, and be treated regularly and systematically.

For this purpose hospital statistics are not suitable, because the cases are not treated from the beginning of the attack. The most suitable material is furnished by the so-called house-physician practice (*hausärztliche Praxis*), that kind of practice in which the physician is employed not only to treat disease after it is developed, but also to prevent its occurrence. In such a practice the cases are seen at the very outset of the attack.

As an exception to all others, Jürgensen's policlinic in Tübingen furnishes substantially these conditions. The military hospitals occasionally furnish statistics of high value. Those of Vogl are especially trustworthy. The following table is composed of the only reliable reports given in the preceding long list:

| | CASES. | DEATHS |
|---|---|---|
| 1. Jürgensen (Tübingen) | 217 | 1 |
| 2. Vogl (Munich) | 221 | 6 |
| 3. Military hospital, Stralsund, 1877–82 | 257 | 1 |
| 4. do in Stettin, 1877–82 | 186 | 3 |
| 5. Brand (Hausärztliche Praxis) | 342 | 1 |
| | 1,223 | 12 |

NOTE: Three cases are excluded from the number reported by Jürgensen, because they were treated with antipyretics.

Although some other military statistics are more favorable, I have selected those given in this last table because the first can be confirmed by reference to the Sanitary Reports, and because the second have occurred, as it were, under my own eye. My own 342 cases cover a period of 30 years, and include all primary complications. An analysis of the 12 fatal cases is as follows:

*Jürgensen.*—1. Treatment begun on the ninth day. Death from perforation and peritonitis.

*Vogl.*—2. Treatment begun on the eighth day. Death from fever.

3. Treatment begun on the sixth day. Death from phlegmonous glossitis.

4. Treatment begun in second week. Death from cardiac weakness.

5. Day of beginning treatment unknown, "already tired for a long time." Death from hemorrhagic diathesis.

6. Treatment begun in the second week. Death from cardiac weakness.

7. Day of beginning treatment not given, temperature 41° C. (195°.8 F.) evidently late in illness. Death from lesion of larynx and œdema of the lungs.

*Stralsund.*—8. Treatment begun on the fourteenth day. Intestinal hemorrhage, diphtheria, petechiæ double hypostatic congestion.

*Stettin.*—9. Treatment begun on the fourteenth day. Pneumonia and intestinal hemorrhage.

10. Treatment begun on the sixth day. Because of inflammation of the joints treatment was stopped.

11. Treatment begun on the sixth day. Death from retropharyngeal abscess.

*Brand.*—12. Treatment begun on the fifth day. Death in a relapse from some unknown cause.

The tenth and twelfth cases do not properly belong in this list. In the others death resulted either from the fever itself or its complications. In 1877, basing the conclusion upon an analysis of 975 cases, I concluded that the treatment must begin before the fifth day; and these 12 cases confirm that conclusion, all cases recovering in which the treatment was begun before the fifth day.

That is to say, the systematic water treatment has, in all cases in which it was employed early and thoroughly, a mortality figure of 0 per cent., and is really a special treatment, as such is and must be. *It is also, therefore, an ideal treatment, since through it the physician is in a position to save the life of the patient with certainty.*

I by no means say that every typhoid fever case treated by the water treatment must recover, but I have said and repeat that those cases in which treatment according to the method of Brand is begun before the fifth day, and is thoroughly carried out, surely recover.

To recapitulate, the mortality under the systematic water treatment will average as follows:

| | | |
|---|---|---|
| *a.* | In "hausärztlichen" practice | 0—1 per cent. |
| *b.* | In military hospitals | 3—4 " " |
| *c.* | In private practice | 3—4 " " |
| *d.* | In general hospitals | 5 per cent. |

At this point in the discussion the author briefly reviews the history of the various methods of treatment of typhoid fever, from the "expectant-symptomatic," in vogue before the fifth decade of the present century, to the present, which he summarizes as follows:

1. Expectant-symptomatic.
2. The old Liebermeister method with all possible variations.
3. The new Liebermeister.
4. The systematic water treatment.
5. The antibacteria method.

He says that the physician is obliged to select from these methods that one which best accomplishes its claims, and asserts that an intelligent choice of method cannot be made upon any other ground.

"Every case treated according to the method of Brand will confirm this principle. Under this treatment it will always happen that—

1. During the action of the baths the process (typhoid) is quiet.
2. At the beginning of an exacerbation it starts up again, and if not combated, continues.
3. With the rise in temperature the functional disturbances appear—a manifest cause and effect.

4. The effect of the bath upon the height of the fever is less than at the beginning.

5. The exacerbation is prevented.

6. The failure to combat the exacerbation brings evils which become more difficult and at times impossible to control, and through repeated failures to combat seem to develop cumulative power. These facts form the basis of the treatment of typhoid fever.

They do not show with a clearness which leaves nothing to be desired just what the treatment of typhoid fever should be, but they furnish an occasional glimpse as to the role which the elevation of temperature plays in the course, process, etc., of the fever.

It therefore follows that, in the treatment of typhoid fever *a*—the severe process should be controlled and the course changed to a light one; *b*—every exacerbation prevented; and *c*—every functional lesion rendered less severe. These demands furnish the indications, which are: *Prevention of every exacerbation, with or without elevation of temperature, throughout the entire illness, day and night, from the beginning to the end.*

It has been already shown above that this can be accomplished by means of the water treatment; and the method is expressed in the formula: *Every 3 hours a cold bath, 15° R. (59° F.) lasting 15 minutes, as long as the temperature rises above 39° C. (102°.2 F.), combined with cold douching and frequently changed compresses over the abdomen.*

Through such baths, repeated regularly at that interval, the exacerbation will be prevented, and the organs rendered capable of their normal functional activity. It is here to be said that only such baths will have this effect which produce abstraction of heat and stimulation. Those which produce only one of these effects will not suffice. When the elevation of the body temperature obstinately remains, the baths must be colder and longer. Vogl, Tripier, Bouveret and Jürgensen repeat the baths at shorter intervals, in such cases, but I do not. If functional disturbances occur at a temperature below 39° C the stimulating treatment is required, viz., tepid baths with cold douching and strong friction.

Of course such conditions as vitium cordis occurring as a primary complication present special situations which must be met. All such complicated situations are fully discussed in my monograph. As is well known, one has less to do in the complicated cases with the typhoid fever than with their *sequelæ*, —tuberculosis pulmonum, cardiac weakness and brain lesions—in short, the increasing functional disturbances of all organs and the accompanying septic, pyæmic and croupous processes. While in an uncomplicated typhoid case the combination of the heat-abstraction with the stimulation is indicated as anti-febrile treatment, in a complicated and degenerated case, stimulation and revulsion are necessary to combat the functional disturbances and local lesions. This condition, or differentiation, is not as yet sufficiently observed, and hence many errors in treatment have arisen. The most common is, that in low temperature three hours after the bath, it is not repeated although severe symptoms on the side of the brain, lungs, heart, and kidneys are present. Of course in such conditions the nature of the bath must be suitable, and the otherwise useful cold bath must not be given. The formula as given applies to the very great majority of cases, and cannot be changed except at the expense of the safety of the result.

The author proposes the question: "Is it possible with the other methods of treatment which obtain to-day to meet the demands in typhoid fever, as well as with the water treatment?" With the "other methods" the author deals summarily. He condemns the symptomatic-expectant treatment upon the ground that since it waits for the rise in temperature before combating it, it is impossible for it to do as well as a method which seeks to prevent its occurrence.

The earlier Liebermeister method is open to a similar objection. The present method of Liebermeister treats the temperature as the most important condition of the typhoid case, which it by no means is, but rather a small part of the process, and its greatest error is the assumption that after the morning remission the course of the process during the day is of no importance. Ziemsen, using this method of Liebermeister, reported the following mortality: For 1882, 10.2%; for 1883, 18.6%. "The author claims that the method of treatment by means of prolonged baths, the method of Riess, is open to the same objections as those of Liebermeister are. In regard to the treatment with thallin the author remarks: 1. A poisoning of the body lasting for 4 or 6 weeks cannot be a desirable thing. 2. Sweatings and rigors are added to the typhoid fever symptoms. 3. That one cannot accomplish much of value in typhoid or febrile diseases by means of a medicine which has only an antipyretic action. 4. The convalescence is protracted (Jacksch, *Verhandlungen des Congress für innere Medicin, 1885*). The author also thinks that the same objections hold good against the use of antipyrin, and he cites the clinical notes of two fatal cases to establish his position.

In regard to the antibacterial or the bacteriacide treatment, the author thinks that it is both not new and not established, having been tried thirty or forty years ago at Hamburg.

The articles close with the following statements: "Jürgensen has thoroughly proved all methods which have appeared during the course of years, and is more and more inclined to return to the systematic water treatment. (*Verhandlungen des Congress für innere Medicin, 1882, p. 102*). At the same congress Liebermeister closed his remarks with the following sentence: "Experience has shown that brilliant results in the treatment of severe febrile cases are obtained only when cold baths are the foundation of the treatment."

ELBERT WING, M. D.

3266 Cottage Grove Ave.

